# Building and Sustaining Effective Partnerships for Training the Next Generation of Global Health Leaders

**DOI:** 10.5334/aogh.3214

**Published:** 2021-07-12

**Authors:** Damalie Nakanjako, Diane Kendall, Nelson K. Sewankambo, Myat Htoo Razak, Bonface Oduor, Theresa Odero, Patricia Garcia, Carey Farquhar

**Affiliations:** 1 Department of Medicine, School of Medicine, College of Health Sciences, Makerere University, Kampala, Uganda; 2 Afya Bora Consortium; 3 Speech & Hearing Sciences, University of Washington, Seattle, USA; 4 Independent researcher, US; 5 International Cancer Institute, Eldoret, Kenya; 6 College of Health Sciences, University of Nairobi, Nairobi, Kenya; 7 Universidad Peruana Cayetano Heredia, Lima, Peru; 8 Departments of Global Health, Medicine and Epidemiology, University of Washington, Seattle, USA

## Abstract

**Introduction:**

Partnerships are essential to creating effective global health leadership training programs. Global pandemics, including the HIV/AIDS pandemic, and more recently the COVID-19 pandemic, have tested the impact and stability of healthcare systems. Partnerships must be fostered to prepare the next generation of leaders to collaborate effectively and improve health globally.

**Objectives:**

We provide key matrices that predict success of partnerships in building global health leadership capacity. We highlight opportunities and challenges to building effective partnerships and provide recommendations to promote development of equitable and mutually beneficial partnerships.

**Findings:**

Critical elements for effective partnership when building global health leadership capacity include shared strategic vision, transparency and excellent communication, as well as intentional monitoring and evaluation of the partnership, not just the project or program. There must be recognition that partnerships can be unpredictable and unequal, especially if the end is not defined early on. Threats to equitable and effective partnerships include funding and co-funding disparities between partners from high-income and low-income countries, inequalities, unshared vision and priorities, skewed decision-making levels, and limited flexibility to minimize inequalities and make changes. Further, imbalances in power, privilege, position, income levels, and institutional resources create opportunities for exploitation of partners, particularly those in low-income countries, which widens the disparities and limits success and sustainability of partnerships. These challenges to effective partnering create the need for objective documentation of disparities at all stages, with key milestones to assess success and the environment to sustain the partnerships and their respective goals.

**Conclusions:**

Developing effective and sustainable partnerships requires a commitment to equality from the start by all partners and an understanding that there will be challenges that could derail otherwise well-intended partnerships. Guidelines and training on evaluation of partnerships exist and should be used, including generic indicators of equity, mutual benefit, and the added value of partnering.

**Key Takeaways:**

- Effective partnerships in building global health leadership capacity require shared strategic vision and intentional monitoring and evaluation of goals
- Inequalities in partnerships may arise from disparities in infrastructure, managerial expertise, administrative and leadership capacity, as well as limited mutual benefit and mutual respect
- To promote equitable and effective partnerships, it is critical to highlight and monitor key measures for success of partnerships at the beginning of each partnership and regularly through the lifetime of the partnership.
- We recommend that partnerships should have legal and financial laws through executed memoranda of understanding, to promote accountability and facilitate objective monitoring and evaluation of the partnership itself.
- More research is needed to understand better the contextual predictors of the broader influence and sustainability of partnership networks in global health leadership training.

## Introduction

Partnerships for global health leadership training are essential to creating effective programs that will prepare future leaders to respond to global health threats illustrated by the HIV/AIDS pandemic and more recently the COVID-19 pandemic. The COVID-19 pandemic has tested the stability of primary and tertiary health care systems globally, challenging national and regional preparedness of health systems, particularly with respect to leadership [1]. Local and regional transformational leadership skills are critical to enhancing preparedness for health emergencies [2] and stronger leadership training is essential for optimal management of global public health crises. Such training must be supported by equitable partnerships, which causes us to re-evaluate the intended and actual impact of various multi-sectoral partnerships that focus on building leadership capacity. This paper describes a spectrum of strategic partnerships that have aimed to equip leaders with skills needed to influence communities for better health. We highlight key challenges and opportunities that influence equitable and mutually beneficial partnerships, and we provide key matrices that are measurable to predict the success of partnerships in building global health leadership capacity. The case study presented illustrates some best practices, opportunities, and recommendations that will guide future partnership endeavors to build capacity for global health leadership training programs.

## Global health leadership training partnerships

**Figure 1:**
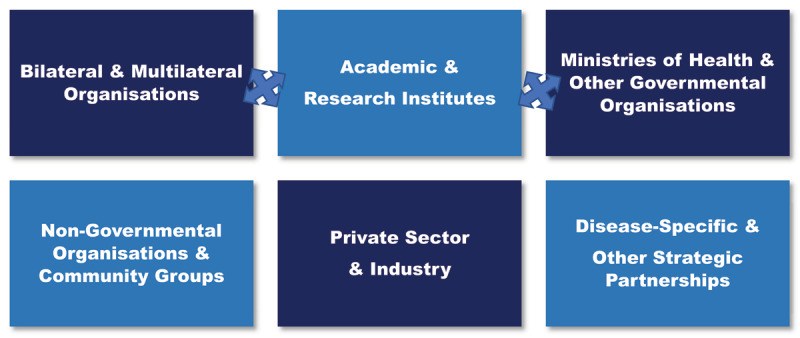
Types of partners that provide opportunities for partnerships.

While effective global health leadership training programs rely on partnerships, these partnerships vary greatly from one another and cover a spectrum from public-private partnerships to those that span academic, bilateral, multilateral, governmental, and non-governmental organizations (***Figure 1***). In the most effective partnerships, both parties agree on shared values and goals for the program or activity. Their shared vision is built on transparent and honest communication that has created trust among those involved, and results in equitable control over the process of achieving the shared vision (***Table 1***). Partners should each benefit from the partnership and efforts need to be made to avoid imbalances resulting from lack of perceived mutual gain. In these ways, effective partnerships are different from many interactions that take place in the global arena, even ones that are highly collaborative. Many collaborations are highly functional, yet there is no engagement of the involved parties at crucial decision-making levels. The relationship may be contractual rather than one of true partnership—where there is joint ownership of the program or activity and each partner contributes and adds value in a unique and equitable way.

**Table 1:** Measures of success of partnerships for global health leadership training*.


KEY ELEMENTS	INDICATORS

**STRATEGIC**	• Shared vision, long-term aims, and clear plans for achieving them • Jointly agreed upon framework of priorities

**HARMONIZED and ALIGNED**	• Consistency of partnership work with local and national plans • Complementary to activities by other development partners

**EFFECTIVE and SUSTAINABLE**	• Delivery of high-quality projects • Achievement of short and long-term milestones

**RESPECTFUL and RECIPROCAL**	• Mutual listening among partners • Cooperative planning, implementation, and learning

**ORGANIZED and ACCOUNTABLE**	• Well-structured management • Transparent decision-making processes

**RESPONSIBLE**	• Activities conducted with integrity and trust

**FLEXIBLE, RESOURCEFUL, and INNOVATIVE**	• Adaptive and responsive to changing circumstances

**COMMITTED to JOINT LEARNING**	• Regular monitoring and evaluation of activities • Reflection on lessons learned • Dissemination of results and knowledge


* Adapted from the THET partnerships for global health.

Defining core principles when building a partnership is an important step in establishing successful partnerships. These principles must be incorporated into the program’s structure and upheld throughout the life of the partnership through constant assessment and evaluation. There is an extensive body of literature on partnerships and several groups have put forward models for building and sustaining effective partnerships in various settings. Tropical Health and Education Trust (THET), an organization that has worked on global health partnerships for decades, puts forward eight principles of effective partnerships in the global health setting [3].

Some potential partners for global health leadership training initiatives are highlighted in ***Figure 1***. The partnerships may take different forms (i.e., public-private; South-South/North-South; disease-specific), largely determined by goal, vision, infrastructure, funding, and key players in the partnerships.

One objective in this paper is to apply these principles to partnership focused on global health leadership training and provide a framework for those developing, re-envisioning, or maintaining and evaluating their partnerships. The opportunities to forge effective partnerships between different organizations and the challenges that are faced are also addressed, including recommendations for evaluation of partnerships at various stages.

## Formation of equitable and effective partnerships for GHL training

GHL training, like any other training, has a ‘hidden’ curriculum. An equitable and effective partnership for GHL training will impact its trainees to appreciate the value of equity. Formation of an equitable and effective partnership for GHL training should start by a clear appreciation that adopting this approach is far from doing anything but business as usual, and requires serious reflections on whether or why having a partnership is essential. What purpose will the partnership serve, and what is its vision? The definitive answers to these questions should be decided and documented by the founding partners after they have come together to have frank conversations on those issues, co-own the decisions, secure commitment, and have a common vision, since this is the solid foundation for an equitable and effective partnership. New partners may join later, but only if they share and buy into what was agreed. This may be revised if a mutually respectful, responsiveness, transparent, trusting relationship is established, and cordial deliberations take place while paying close attention to the centrality of distributive justice.

The recent development, initiation, and adoption of the research fairness initiative (RFI) is an important signpost towards formation and continuing attention in management for continuous improvement and sustainability of an equitable and effective partnership [4] The RFI aims to create transparency, enabling partners to negotiate before partnerships happen, increasing trust and a sense of co-ownership, and building a growing evidence base of best practices, guidelines, and standards. Similarly, the increasing demand to demonstrate impact of leadership training on prevailing approaches to global health issues emphasizes the need to pay attention to the intended outcome and impact of partnerships right from inception.

The World Health Organization Tropical Disease Research program (WHO/TDR) has taken the first step in utilizing the RFI and has found it valuable in assessing its performance as an equitable organization. The goal of an equitable and effective partnership for a GHL training program is one that requires planning, as well as continuous nurturing and management. As part of that process, there is need for negotiation and renegotiation of everything, and attention to detail, especially around participation in governance, financial, and scientific decision making.

Funders have a major role to play in building equitable and effective partnerships. They have received increasing attention by the United Kingdom Collaborative on Development Research (UKCDR) in its report “Building Partnerships of Equals: Role of Funders in Equitable and Effective International Development Research Collaborations [5].” This report emphasizes the role that funders can play in building partnerships. Key among the recommendations is that funders need to recognize the time and costs of building international collaborations.

No doubt there is not a one-size-fits-all approach to developing equitable partnerships. The approach to formation of partnerships will vary depending on several factors, including context. The COVID-19 pandemic has clearly re-awakened our need for GHL training, which should be an essential component for epidemic and pandemic preparedness and response.

## Sustaining an equitable and effective global health partnership

Sustaining an equitable and effective global health partnership requires analytical, forward-thinking, and soul-searching efforts from all stakeholders, including governmental and funding partners. Improvements and advances in global health partnership in the last few decades have been made possible through increasing and active participation of global health workers from low-and-middle income countries (LMIC) in leadership and decision-making. This has been complemented by better collaboration and support from “traditional” decision-makers and funders such as governmental agencies, multi-lateral agencies, foundations, and academic institutions based in high-income countries (HIC). Training and collaboration on global HIV/AIDS, chronic diseases, and health systems research have propelled many leaders from LMIC and HIC to strengthen collective leadership across countries and institutions as well as build leadership capacity and partnerships within countries [6,7]. Global health partnerships must continue to improve, strengthen, support, and prepare future generations of leaders to collaborate effectively in making the world a healthier place for everyone. Successful partnerships have birthed self-sustaining centers of excellence in Africa such as the Noguchi Memorial Institute for Medical Research in Ghana, Infectious Diseases Institute at Makerere University in Uganda, and the Academic Model Providing Access to Healthcare (AMPATH) in Kenya [8], which are playing leading roles in responding the HIV/AIDS and COVID-19 global pandemics as well as other local and regional health emergencies. To sustain partnerships that are equitable and built with a common vision, the following key practical approaches should be implemented:

- Ensure equal participation and decision-making platforms for representatives from the affected/intended countries/communities, national/global/regional experts, governmental agencies, and funding partners in strategy development of global health projects (including training projects).
- Promote and practice equitable participation of inter-generational, gender, and inter-professional groups of affected/intended countries in strategy development, implementation, and monitoring for timely, effective, and sustainable global heath projects.
- Maintain regular communications and interim progress reports from the project to other key stakeholders for transparency and timely interventions for successful projects.
- Reassess the direction, progress, and impact of the project at least one year before the initial end of the funding period and develop appropriate action plan in moving forward (e.g., additional funding, revision of the project direction, transition, or conclusion of the project) in collaboration with key stakeholders.

## Assessment of partnership effectiveness and equity

Partnerships form because one organization or group is not able to accomplish something on its own. Global health leadership training is dependent on effective partnerships because a robust program requires the diverse sets of opportunities that are only available across multiple different organizations. Leadership competences and skills are assimilated and applied best when learned within a specific context. For example, the African Research Coalition for Health (ARCH), a network of 11 African-led consortia covering 54 African research institutions and universities across 17 sub-Saharan countries, was formed in the context of capacity building for research leadership, and has produced leaders in the sub-Saharan African response to emerging infections of global health importance, such as SARS-CoV-2 [2]. Therefore, the principle that focuses on partnerships being harmonized and aligned is especially important. The partnership must be working on a program that is consistent with local and national plans and complements the activities of other development partners. While this principle will be important for all partnerships, it is likely to be more important for global health leadership training than for other types of partnerships. A second critical component of leadership training is that trainees learn what it means to be part of a team, lead a team, and work with a team approach. The partnership needs to model this behavior by listening to each other, learning together, planning, implementing, and adapting the program with input from all partners. Measuring success on these parameters is challenging but prioritizing specific aspects of the partnership can be done and iterative improvements will be possible when parties appreciate the partnership’s value and are committed to sustaining it.

As Paul Farmer highlights, partnerships can be unpredictable and unequal, especially if the end is not defined at the beginning [9]. Unfortunately, investigators are often more interested in establishing the implementation and output of the programs than measuring the quality of the relationships and identifying predictors to successful partnerships. In a rapid evidence review of published papers and gray literature relating to the effectiveness of working in partnership, Kelly *et al*. found that evidence for the effectiveness of health partnerships was scanty both in terms of quantity and quality. One recommendation is that administrators need guidelines and training to develop generic indicators and objectively monitor and evaluate benefits and challenges that the partnership is facing [10] ***Table 1*** outlines some key questions about global health leadership training partnerships, based on the THET, which emphasizes specific attributes, structures, and milestones that can be measured objectively to monitor effectiveness and impact of global health partnerships [3]. Given their broad spectrum, the measures can be monitored using both quantitative and qualitative methods, during the planning and execution of global GHL training programs.

**Case Study: International Cancer Institute (ICI) Kenya—Center for Creative Leadership (CCL) Belgium strategic partnership.** This is a case study of a dyad partnership of not-for-profit organizations, ICI and CCL. ICI provides training for cancer health care professionals in sub-Saharan Africa (SSA) and CCL provides training in leadership development. They partnered with a common goal to build leadership capacity in primary cancer care through equitable engagement for sustained impact and mutually beneficial relations. The roles of each partner were clearly defined at formation of the partnership. While ICI provided the training infrastructure and funding to facilitate faculty, curriculum development, and training materials, CCL provided faculty training and tailor-made curriculum through their flagship Leadership Development Program (LDP). Both partners facilitated the mentorship programs through webinars and personalized video follow up calls.

ICI funds the program, contrary to the norm, where funding is provided by the partner in high income countries. This presents a shift in partnership dynamics and fostered equity among the two partners despite the huge economic inequalities between the two organizations based on their economic and geographical standpoint.

**Key successes** have been a robust virtual mentorship program, harnessing technological advances of webinars and personalized video follow up calls, and flexible and robust training curricula that have made both organizations nimble in offering training programs in the face of the global COVID-19 pandemic.

## Opportunities and challenges posed by GHL training programs

Global health partnerships address global inequities and provide opportunities for skills building, technology transfer, and resource sharing between partner institutions [11,12]. Starting a partnership requires a considerable reflection on the challenges and threats that could derail an otherwise well-intended partnership, some of which are outlined below.

- **Burden to weak partners to keep up with the demands of the partnership:** Okeke *et al*. emphasized the burden global health partnerships can place on the local institutions in terms of local proposal review of projects, demand for office and desk space, and overwhelming laboratory services with equipment that need electricity as well as the need to test normal control subjects [13]. Such an imbalance of power, privilege, and positionality due to differences in income levels, education levels, socio-cultural differences, and institutional resources creates a feeling of partner exploitation to the weak partner who fails to cope and the strong partners who may feel low return on hefty investments to uplift the weak partner [13]. This challenge is likely a consequence of inadequate attention to the inequalities in infrastructure, managerial expertise, administration, and leadership at the planning and formation stage of the partnerships (***Table 1***) [14].
- **Uncertain sustainability:** Partners often struggle to sustain GHL training programs beyond the formal duration of the partnerships, likely because they remain ‘strained’ from the struggle to sustain the demands of a partnership within an already constrained environment, which Okeke terms as “harm to individuals and organizations long after the HIC partner leaves.” This often leads to attrition of the trainees if they cannot take on or sustain the project tasks after the HIC partner leaves [14].
- **External threats to good partnerships:** Funding mechanisms occasionally influence the partners involved in the respective type of partnerships and the funding restrictions occasionally contribute to widening rather than closing the inequalities among partners. Co-funding requirements may restrict engagement of more vulnerable partners that are unable to mobilize co-funds. Unequal contributions by otherwise equal partners can also cause tension and dissolution of partnerships. For example, if the majority of funds for a strategic GHL come from one institution, trying to create an equitable partnership may be challenging. Recognition of such limitations at the beginning is critical to inform strategic capacity building including financial management systems, to reduce the inequalities that may hinder the achievement of the well-intended partnerships.
- **Lack of Equity:** Inherently, global health academic partnerships typically present with unequal power relationships between nations, people, and institutions [10], and this underscores the need to nurture equitable engagements and foster impactful, ethical, and mutually beneficial relationships [15]. It is critical to evaluate equity of key structures such as funded staff, leadership capacity, space, funding, and systems to promote growth and absorption of global health leadership trainees and graduates in order to produce the desired impact. Highlighting inequalities in such key aspects would enable the partnerships to plan for strategies to bridge rather than widen the inequalities. Hence the need for objective documentation of disparities at all stages of the partnerships, with key milestones to assess success and the environment to sustain the success.

## Conclusion

Strong global health leadership is critical for an immediate, effective, and impactful public health response, especially during emergent and overwhelming crises such as the COVID-19 pandemic. We emphasize that the best way to build successful, transformative global health leadership is through training programs that embrace the principles and practices of equitable partnerships. The formation, monitoring, and sustainability of the partnership and all aspects of the partnership require constant vigilance and review. When created and nurtured correctly, equitable and mutually beneficial partnerships play a critical role in the success of global health leadership training programs and are key to building a cadre of global health leaders capable of tackling the many current and future public health challenges.
